# Plasminogen kringle 5 suppresses gastric cancer via regulating HIF-1*α* and GRP78

**DOI:** 10.1038/cddis.2017.528

**Published:** 2017-10-26

**Authors:** Shuhuan Fang, Honghai Hong, Lei Li, Dan He, Zumin Xu, Shaoyuan Zuo, Jing Han, Qiyuan Wu, Zhiyu Dai, Weibin Cai, Jianxing Ma, Chunkui Shao, Guoquan Gao, Xia Yang

**Affiliations:** 1 DME Center, Clinical Pharmacology Institute, Guangzhou University of Chinese Medicine, Guangzhou, China; 2 Department of Biochemistry, Zhongshan School of Medicine, Sun Yat-sen University, Guangzhou, China; 3 Department of Reproductive Medicine Center, Key Laboratory for Reproductive Medicine of Guangdong Province, Third Affiliated Hospital of Guangzhou Medical University, 63 Duobao Road, Guangzhou 510150, China; 4 Department of Pathology, The Third Affiliated Hospital, Sun Yat-sen University, Guangzhou, China; 5 Cancer Center, Affiliated Hospital of Guangdong Medical College, Zhanjiang, China; 6 Department of Biochemistry, Basic Medical College, Dali College, Dali, China; 7 International Department, The Affiliated High School of South China Normal University, Guangzhou, China; 8 Department of Physiology, University of Oklahoma Health Sciences Center, Oklahoma City, Oklahoma, USA; 9 China Key Laboratory of Tropical Disease Control (Sun Yat-sen University), Ministry of Education, Guangzhou, China; 10 Guangdong Engineering & Technology Research Center for Gene Manipulation and Biomacromolecular Products (Sun Yat-sen University), Guangzhou, China

## Abstract

Inhibition of tumour angiogenesis has an important role in antitumour therapy. However, a recent study indicates that antiangiogenesis therapy may lead to glucose-related protein 78 (GRP78) associated antiapoptotic resistance. The present study aims to elucidate the dual effects of plasminogen kringle 5 (K5) on tumour angiogenesis and apoptosis induction by targeting hypoxia-inducible factor 1*α* (HIF-1*α*) and GRP78. Co-immunoprecipitation and western blotting were used for examining the ubiquitination of HIF-1*α* and analysing angiogenesis and apoptosis-associated proteins. K5 promoted the sumo/ubiquitin-mediated proteasomal degradation of HIF-1*α* by upregulating von Hippel-Lindau protein under hypoxia, resulting in the reduction of vascular endothelial growth factor and thus suppressing tumour angiogenesis. Furthermore, K5 decreased GRP78 expression via downregulation of phosphorylated extracellular-regulated protein kinase, leading to caspase-7 cleavage and tumour cell apoptosis. Blocking voltage-dependent anion channel abrogated the effects of K5 on both HIF-1*α* and GRP78. K5 significantly inhibited the growth of gastric carcinoma xenografts by inhibiting both angiogenesis and apoptosis. The dual effects suggest that K5 might be a promising bio-therapeutic agent in the treatment of gastric cancer, particularly in patients who exhibit the induction of GRP78.

Gastric cancer is an aggressive malignancy that is frequently diagnosed at an advanced stage with poor prognosis. Although surgery and/or a combination of chemotherapy improve the survival rates, the 5-year relative survival rates of the patients receiving these treatments remains low at 30% and that of the patients with advanced disease is <1 year.^1, 2^ Therefore, it is necessary to develop more effective therapeutic strategies.

As the growth and survival of most tumour cells is dependent on oxygen and nutrient supply, angiogenesis has been a long-standing and attractive therapeutic target for treating malignant tumours.^3^ Angiogenesis is induced by hypoxic conditions and regulated by hypoxia-inducible factor 1*α* (HIF-1*α*) and its major target gene, vascular endothelial growth factor (VEGF).^4^ The expression of HIF-1*α* and VEGF is increased in many human cancers.^5, 6^ Close association between high-level expression of HIF-1*α* and VEGF and patient mortality have been demonstrated in cancers of brain, breast, cervix, oropharynx, ovary, and uterus.^7^ Preclinical studies have shown that inhibition of VEGF pathway impedes tumour growth. VEGF-neutralizing antibody Avastin (bevacizumab) and VEGF receptor tyrosine kinase inhibitors (sorafenib and sunitinib) inhibit primary tumour growth in clinical applications and have been used as anticancer treatments in several tumour types.^8^ However, clinical observations indicate that these therapies may have limited efficacy because the response has been mostly transient, in addition to the development of drug resistance.^9^ Glucose-related protein 78 (GRP78) could be induced by severe glucose and oxygen deprivation resulting from antivascular and antiangiogenesis therapies, which could lead to drug resistance in an HIF-1*α*-independent manner.^10^ In conditions of stress or hypoxia associated with oncogenesis, GRP78, a major endoplasmic reticulum chaperone, has an essential role in counteracting the apoptosis, which promotes cancer cell proliferation, survival, metastasis, and resistance to a wide variety of therapies.^11, 12^

K5, the fifth kringle domain in human plasminogen, has been shown to display the most potent inhibitory effect on endothelial cells and angiogenesis.^13^ Some studies have demonstrated the antiangiogenesis and antitumour effects of K5 both *in vivo* and *in vitro*.^14, 15^ Our previous study also showed that downregulation of HIF-1*α* by K5 is responsible for the decreased VEGF expression in endothelial cells and lung cancer cells.^16^ Furthermore, K5 has been shown to directly induce apoptosis and cause cell cycle arrest in proliferating endothelial cells and stressed tumour cells through cell surface voltage-dependent anion channel (VDAC)^17, 18^ or GRP78.^19, 20^ However, the receptors and the downstream signals by which K5 downregulates HIF-1*α* and GRP78 in cancer cells have remained unknown. The present study was designed to investigate the effects and the mechanism underlying these effects of K5 on both HIF-1*α*-induced angiogenesis and GRP78-dependent apoptosis resistance in gastric cancer cells.

## Results

### High expression levels of CD34, HIF-1α, VEGF, and GRP78 in human gastric cancer

**Figure 1:**
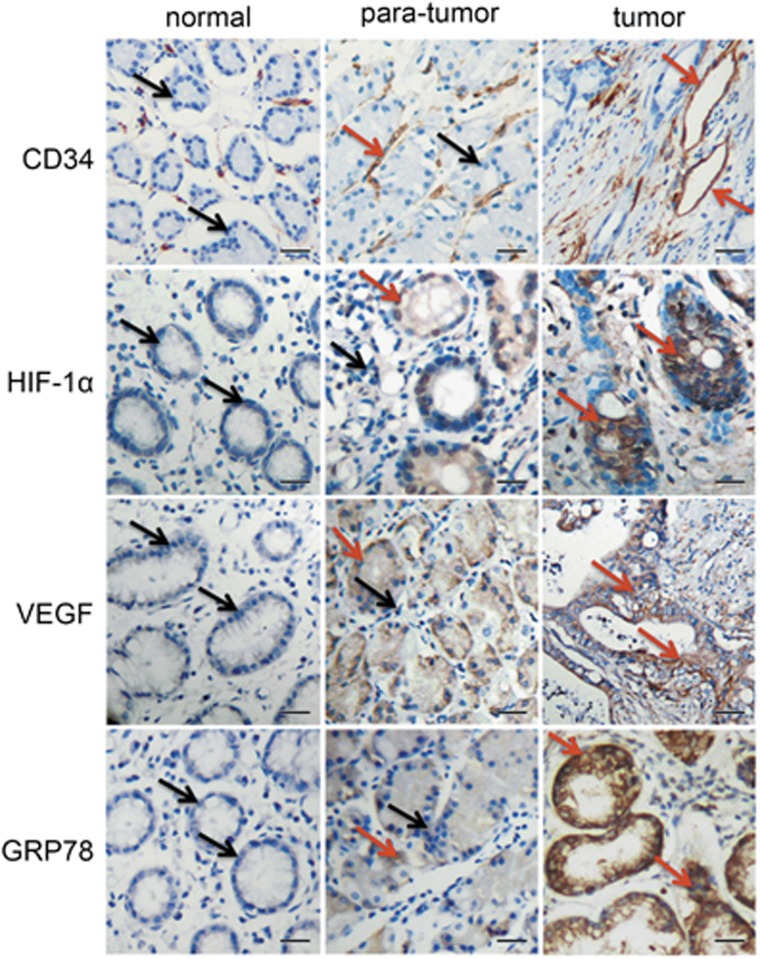
Expression of CD34, HIF-1*α*, VEGF, and GRP78 in normal human gastric tissues, para-tumour tissues, and tumour tissues. Immunohistochemical staining of paraffin-embedded serial tissue sections from 30 normal gastric tissue specimens, 17 gastric para-tumour specimens, and 42 gastric carcinoma specimens, with antibodies against CD34, HIF-1*α*, VEGF, and GRP78. Immunoreactivity is indicated by brown staining. The red arrow represent positive, and the black arrow represent negative. Scale bars represent 20 *μ*m

To evaluate angiogenesis-related molecules and antiapoptosis molecule, GRP78, in gastric cancer, we first examined microvessel density (MVD; CD34), HIF-1*α*, VEGF, and GRP78 in 42 cases of gastric carcinoma. The clinical–pathological characteristics observed in immunohistochemical analyses are shown in Figure 1a. The levels of CD34 and HIF-1*α* in the tumour and para-tumour groups and in the tumour and normal groups are significantly different (Table 1). The difference in VEGF expression between tumour and normal groups is statistically significant, whereas there was no significant difference between tumour and para-tumour groups and between para-tumour and normal groups. The differences in the expression of GRP78 between tumour and normal groups and between tumour and para-tumour groups are statistically significant, whereas there was no significant difference between normal and para-tumour groups (Table 1). These results suggested that tumour angiogenesis was stimulated by HIF-1*α* and VEGF, and GRP78 may facilitate human gastric cancer growth.

### K5 inhibits angiogenesis in heterotopic tumours in athymic mice, resulting from human gastric cancer transplants

**Figure 2:**
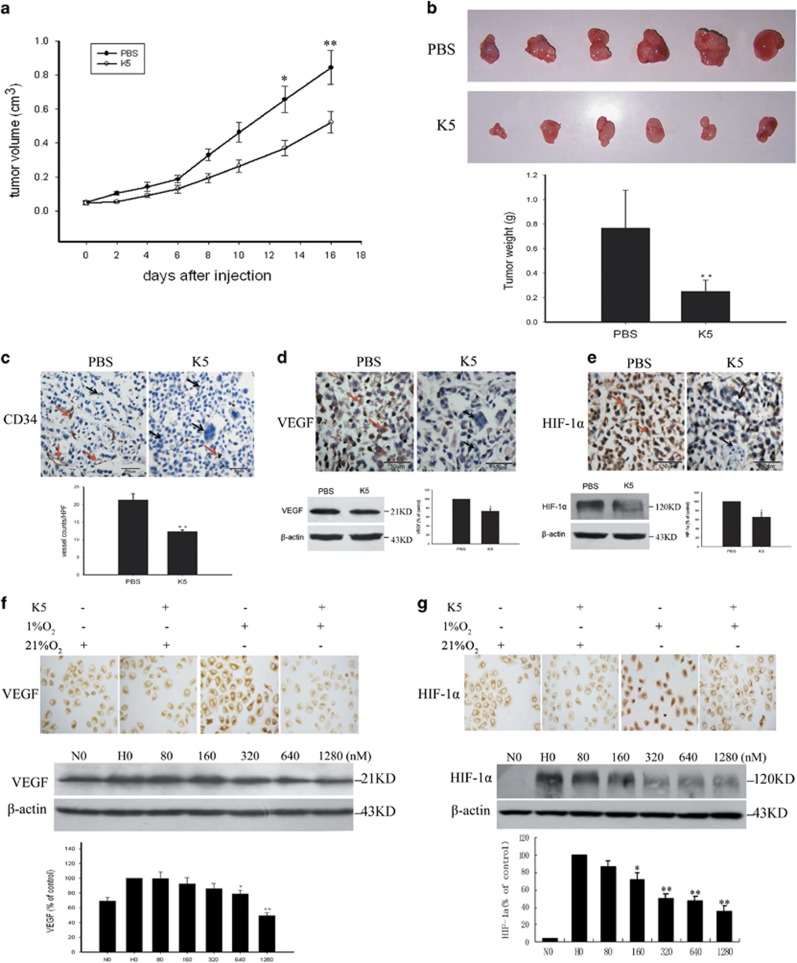
K5 suppresses human gastric cancer growth and downregulates HIF-1*α* and VEGF. (**a**) Kinetics of tumour growth. Data are presented as mean±S.E.M., *P
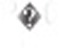
0.05, **P<0.01. (**b**) Tumours were collected and weighed on day 22 after transplantation. An average suppression of 67% of primary tumour growth was observed in the K5-treated group *versus* PBS-treated group. **P<0.01. (**c**) Microvessel density was assessed by immunohistochemical staining for CD34, a marker of endothelial cells. Number of microvessels was counted from five randomly selected fields. **P<0.01. (**d** and **e**) Immunohistochemistry and western blotting were used to detect the expression of VEGF (**d**) and HIF-1*α* (**e**) in mice tumour tissues. Corresponding quantifications by western blotting are presented as mean±S.E.M.; *n*=3, *P<0.05. Scale bars represent 50 *μ*m. (**f** and **g**) SGC-7901 cells were treated with K5 (640 nM for immunocytochemistry and 0–1280 nM for western blotting) under normoxia (21% O_2_) or hypoxic (1% O_2_) conditions for 12 h. The expression of VEGF (**f**) and HIF-1*α* (**g**) was determined by immunocytochemistry and western blotting analysis. Corresponding semiquantification values measured by western blotting densitometry are presented as mean±S.E.M., *n*=3, *P<0.05, **P<0.01. N, normoxia; H, hypoxia

To evaluate the effect of K5 on tumour growth, heterotopic tumour xenografts of human gastric cancer were established in athymic mice. The animals received intraperitoneal injection of K5 or PBS and the tumour growth was monitored (Figure 2a). Statistical analysis indicated that tumour weight was significantly lower in the K5-injected mice compared with that in the PBS-treated mice. The mean suppression of primary tumour growth in the K5-treated group was 67% (*P*<0.01, *n*=6, Figure 2b). To understand the mechanism underlying the suppression of tumour growth, tumour sections were analysed for MVD by staining with anti-CD34 antibody. In contrast to the PBS group, K5-treated group showed a dramatic reduction in MVD (*P*<0.01; Figure 2c).

### K5 downregulates the expression of HIF-1α and VEGF *in vivo* and *in vitro*

A large body of data indicates that HIF-1*α* and VEGF contribute to tumour angiogenesis. To determine whether HIF-1*α*-VEGF signalling pathway has a role in tumour angiogenesis, tumour sections were analysed for VEGF and HIF-1*α*. The results of immunohistochemical staining and western blotting analysis (Figures 2d and e) showed that the expression of VEGF and HIF-1*α* were downregulated by K5. To further explore the effect of K5 on VEGF and HIF-1*α*, *in vitro* experiments were performed using SGC-7901. The results indicated that the expression of VEGF and HIF-1*α* in SGC-7901 cells was upregulated under hypoxic conditions. K5 markedly suppressed the expression of VEGF and HIF-1*α* in a dose-dependent manner (Figures 2f and g). The downregulation of VEGF does not contribute to the pro-apoptotic effects of K5, because there are no VEGF receptors (Flt-1 and KDR) on SGC-7901 cells (Supplementary Figure S1). These data suggested that K5 inhibited the neovascularization of human gastric cancer by downregulating the VEGF and HIF-1*α* expression.

### K5 promotes the SUMO/ubiquitin-mediated proteasomal degradation of HIF-1α

**Figure 3:**
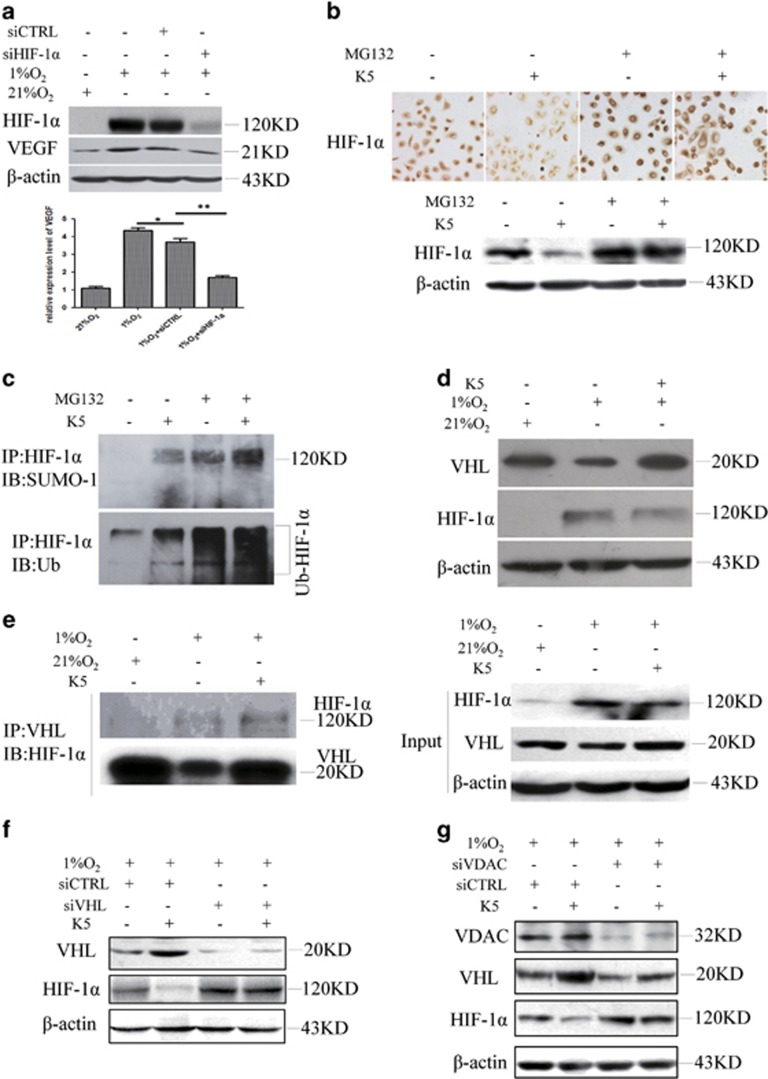
K5 promotes HIF-1*α* protein degradation via the VHL–proteasome pathway resulting in the inhibition of VEGF paracrine response. (**a**) VEGF is regulated by HIF-1*α* in SGC-7901 cells. SGC-7901 cells were transfected with the vector encoding a short hairpin (sh) RNA targeting HIF-1*α* or a scrambled control for 24 h and then exposed to 1% O_2_ or 21% O_2_ for another 24 h. The protein levels of HIF-1*α* and VEGF were measured. (**b**) SGC-7901 cells were cultured with or without 640 nM K5, followed by treatment with proteasome inhibitor, MG132 (5 *μ*M), for 10 h under hypoxia. The expression of HIF-1*α* was detected by immunocytochemistry (Up) or western blotting assay (Down). (**c**) SGC-7901 cells were cultured with or without K5 (640 nM) and MG132 (5 *μ*M) for 10 h under hypoxia. Co-IP was performed with anti-HIF-1*α* antibody and was analysed by western blotting specific to SUMO (Up) and ubiquitin (Down). (**d**) SGC-7901 cells were treated with 640 nM K5 for 8 h and VHL expression was then assessed by western blotting. (**e**) SGC-7901 cells were treated as in panel (**c**), and cell lysates were precipitated with antibody against VHL and analysed by western blotting specific to HIF-1*α*. (**f**) SGC-7901 cells were transfected with vector encoding a short hairpin (sh) RNA targeting VHL or a scrambled control for 24 h and then treated with 640 nM K5 under 1% O_2_ for 15 h. The protein levels of VHL and HIF-1*α* were measured. (**g**) Immunoblotting assays of VDAC and HIF-1*α* protein in SGC-7901 cells transfected with siRNA specific to VDAC or a scrambled control (siCTRL) for 24 h and then cultured with or without 640 nM K5 for another 10 h under hypoxia

To investigate whether VEGF is regulated by HIF-1*α* in SGC-7901 cells, specific siRNA targeting HIF-1*α* was used. Knockdown of HIF-1*α* inhibited the accumulation of VEGF protein dramatically (Figure 3a). We found that HIF-1*α* accumulated significantly after MG132 treatment (Figure 3b). Moreover, the downregulation of HIF-1*α* by K5 under hypoxia was blocked by MG132 (Figure 3b). These observations suggested that the downregulation of HIF-1*α* by K5 may be through ubiquitin–proteasomal degradation pathway. Recent data showed that SUMOylation of HIF-1*α* facilitated polyubiquitination, leading to the proteasomal degradation of HIF-1*α*.^21^ To examine whether K5 has the same role in this process, HIF-1*α* was precipitated by a specific anti-HIF-1*α* antibody. We observed that K5 promoted the SUMOylation and ubiquitination of HIF-1*α*, which eventually resulted in its degradation (Figure 3c).

### K5 increases VHL expression and promotes HIF-1α/VHL binding via VDAC

Our results also showed that K5 upregulated von Hippel-Lindau protein (VHL) (Figure 3d), which could bind to HIF-1*α* and function as an E3 ubiquitin ligase in the proteasomal degradation of HIF-1*α* under hypoxia.^22, 23^ To further confirm the specific contribution of VHL to K5-induced proteasomal degradation of HIF-1*α*, co-immunoprecipitation (co-IP) assay and VHL-specific knockdown were performed. The results showed that K5 promoted the binding of VHL to HIF-1*α* (Figure 3e). Furthermore, the effect of K5 on HIF-1*α* was abolished when VHL was specifically knocked down (Figure 3f). However, we found no changes in prolyl hydroxylase domain proteins 1 (PHD1) and PHD3. PHD2 did exhibit an increase but the increase was not statistically significant (Supplementary Figure S2), suggesting VHL, rather than PHDs, could be the target of K5. Earlier studies have proposed VDAC as the receptor of K5 in K5-induced endothelial cell apoptosis.^17, 18^ We therefore tried to demonstrate the role of VDAC in VHL regulation. Our results showed that specific knockdown of VDAC using siRNA abrogated the induction of VHL by K5 (Figure 3g). Taken together, these data strongly suggest that K5 promoted the SUMO/ubiquitin pathway-mediated proteasomal degradation of HIF-1*α* by inducing VHL and binding to it, which is mediated by VDAC.

### K5 has a growth-suppressive and pro-apoptotic effect on SGC-7901 cells under hypoxia

**Figure 4:**
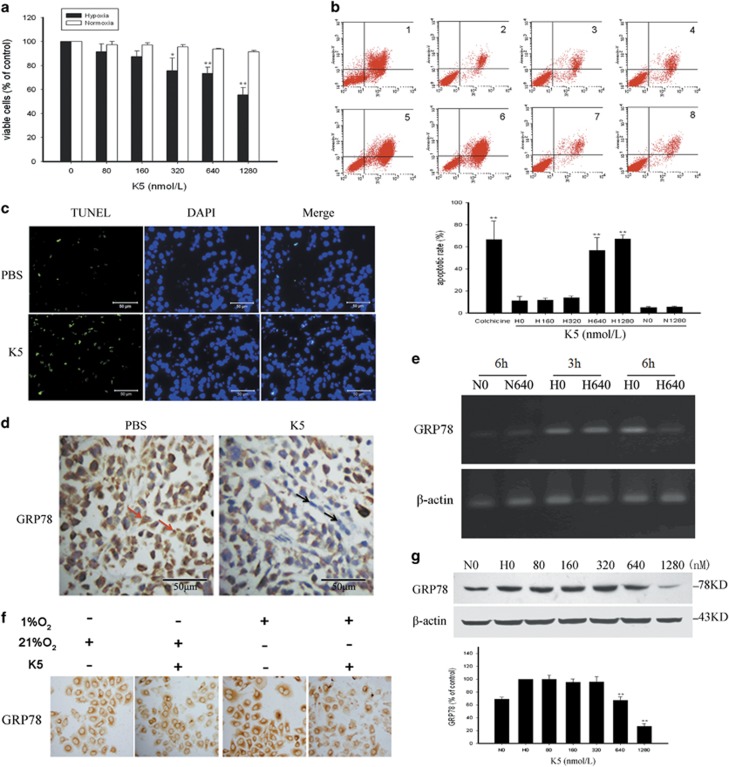
K5 inhibits the proliferation of SCG-7901 cells and suppresses the expression of GRP78. (**a**) SGC-7901 cells were treated with the indicated concentrations of K5 for 72 h under normoxia (□) or hypoxia (▪). The viable cells were quantified by MTT. (**b**) SGC-7901 cells were treated with K5 (0, 160, 320, 640, or 1280 nM) for 48 h. Cells treated with 25 *μ*M colchicine served as positive control. Apoptotic cells were quantified by flow cytometry. Data are shown as mean±S.E.M.; *n*=3, **P<0.01. (**c**) Frozen tumour sections were subjected to TUNEL assay (green), and the nuclei were counterstained with DAPI (blue) to identify apoptotic cells. Scale bars represent 50 *μ*m. (**d**) Immunohistochemistry was used to detect GRP78 expression in mice tumour tissues. Scale bars represent 50 *μ*m. (**e**) SGC-7901 cells were cultured with or without 640 nM K5 under normoxia or hypoxia for 3 or 6 h. GRP78 mRNA levels were analysed by reverse transcriptase-PCR. (**f** and **g**) K5 treated SGC-7901 cells were exposed to 1% or 21% O_2_ for 15 h and GRP 78 expression was analysed by immunocytochemistry (**f**) and western blotting (**g**). The corresponding semiquantifications by western blotting densitometry are presented as mean±S.E.M., *n*=3; **P<0.01. N, normoxia; H, hypoxia

3-[4,5-Dimethylthiazol-2-yl]-2,5-diphenyl tetrazolium bromide (MTT) assay was used to gain insights into the antiproliferative effect of K5 on SGC-7901 cells. K5 inhibited SGC-7901 cell proliferation in a dose-dependent manner under hypoxia, whereas it had no effect under normoxia (Figure 4a). Moreover, flow cytometric analysis showed that K5 induced apoptosis of SGC-7901 cells under hypoxia but not under normoxia (Figure 4b). These results suggest that K5 has a growth-suppressive and pro-apoptotic effect on SGC-7901 cells under hypoxia, which is consistent with the results of a TUNEL assay, which demonstrated that K5 induced apoptosis in xenograft gastric carcinoma (Figure 4c). Similar result was obtained in another gastric cancer cell type, BGC-823 (Supplementary Figure S3).

### K5 downregulates GRP78 *in vivo* and *in vitro*

GRP78 is induced in a wide variety of cancer cells and has an important role in inhibiting apoptosis.^12^ Our results showed that GRP78 was downregulated by K5 in the xenografts (Figure 4d). The effect of K5 on GRP78 expression was further confirmed by RT-PCR. The result showed that GRP78 mRNA was decreased in a time-dependent manner (Figure 4e). Consistent with the changes in mRNA levels, both immunocytochemistry and western blotting analysis indicated that the protein level of GRP78 was upregulated under hypoxic condition in SGC-7901 cells and reduced significantly by K5 in a dose-dependent manner (Figures 4g and g).

### Pro-apoptotic effect of K5 is mediated via downregulation of GRP78 in SGC-7901 cells under hypoxia

**Figure 5:**
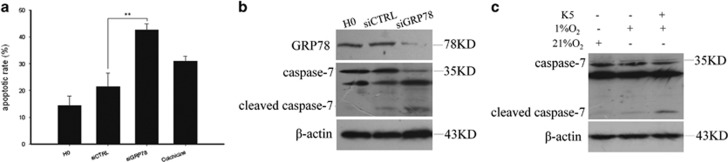
Pro-apoptotic effect of K5 is mediated via downregulation of GRP78 in SGC-7901 cells under hypoxia. (**a** and **b**) SGC-7901 cells were transfected with siRNA specific to GRP78 or a scrambled control (siCTRL) for 24 h under normoxia and then incubated in hypoxic conditions for another 24 h. Apoptotic cells were quantified by flow cytometry. Colchicine treatment constituted positive control. **P<0.01 (**a**). Protein levels of GRP78 and caspase-7 were measured by western blotting (**b**). (**c**) SGC-7901 cells cultured with or without 640 nM K5 were exposed to 1% or 21% O_2_ for 24 h prior to western blotting analysis of caspase-7 levels in total cell lysates.

To confirm the relevance of the effects of K5 to apoptosis and GRP78, GRP78 was knocked down using specific siRNA. We observed that GRP78 knockdown significantly increased the apoptotic rate in SGC-7901 cells (Figure 5a), accompanied by the cleavage of pro-caspase-7 (Figure 5b). K5 induced the cleavage of pro-caspase-7 (Figure 5c). These data confirm that the pro-apoptotic effect of K5 is mediated via downregulation of GRP78 in SGC-7901 cells under hypoxia.

### K5 downregulates GRP78 via VDAC and ERK phosphorylation

**Figure 6:**
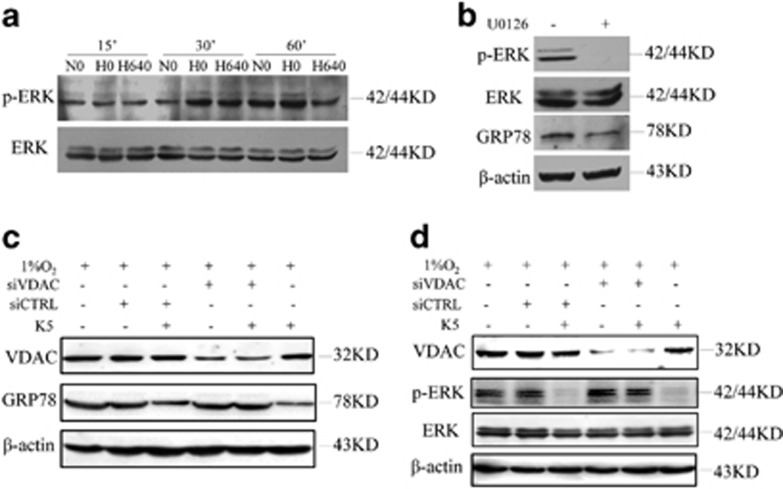
K5 downregulates GRP78 via VDAC and ERK phosphorylation. (**a**) K5 downregulates p-ERK expression. SGC-7901 cells were cultured with or without 640 nM K5 for 15, 30, or 60 min under normoxia (N) or hypoxia (H). The protein level of p-ERK and ERK were detected by western blotting. (**b**) Immunoblot assays of p-ERK, ERK, and GRP78 proteins from SGC-7901 cells cultured in the presence of 10 *μ*M U0126 for 12 h. (**c** and **d**) VDAC is primarily responsible for the downregulation of p-ERK and GRP78 expression by K5. Immunoblotting assays of VDAC, GRP78, p-ERK, and ERK proteins from SGC-7901 cells transfected with siRNA specific to VDAC or a scrambled control (siCTRL) for 24 h and then treated with 640 nM K5 for another 24 h (**c**) or 60 min (**d**) under hypoxia

To identify the possible additional signalling pathways involved in the apoptosis induced by K5, the total protein levels and the phosphorylation status of proteins involved in GRP78 synthesis were examined. The results showed that K5 downregulated p-ERK at 1 h (Figure 6a). We then used U0126 to evaluate the roles of p-ERK and GRP78. The results indicated that U0126 suppressed p-ERK protein expression, along with the downregulation of GRP78 (Figure 6b). In addition, neither Jun N-terminal kinase (JNK) nor p38 was necessary for the K5-induced apoptosis (Supplementary Figure S4). VDAC was specifically knocked down to validate the role of VDAC in the process of K5-mediated downregulation of GRP78. We found that specific knockdown of VDAC prevented the downregulation of GRP78 by K5 (Figure 6c). Furthermore, the downregulation of p-ERK was also abolished by siVDAC (Figure 6d). The results of all these experiments together demonstrate that VDAC has a vital role in K5-induced gastric cell apoptosis via the p-ERK–GRP78 pathway.

## Discussion

In the present study, we confirmed the antitumour activity of K5, which significantly inhibits gastric cancer growth via the dual effects of antiangiogenesis and pro-apoptosis. Importantly, we found that K5 promoted the SUMO/ubiquitin-mediated proteasomal degradation of HIF-1*α* by inducing VHL expression, resulting in the inhibition of the paracrine effect of VEGF. Our results showed for the first time that the pro-apoptotic effect of K5 was mediated via downregulation of GRP78. Our results also showed that VDAC, as a critical signalling receptor of K5, mediated the dual effects of antiangiogenesis and pro-apoptosis of K5.

Tumour angiogenesis is the core process in tumourigenesis. One of the major drivers of tumour angiogenesis is hypoxia, which characterizes the tumour microenvironment. At the molecular level, the master switch orchestrating the cellular response to low O_2_ environment is generally considered to be the transcription factor HIF, which acts as the angiogenic switch and increases the formation of pro-angiogenic factors, such as VEGF.^24, 25^ Some studies have indicated that gastric carcinoma patients with VEGF-positive tumours had worse prognosis than those with VEGF-negative tumours.^5, 6^ We examined in this study gastric carcinoma tissues from 42 patients and found that CD34 (MVD), VEGF, and HIF-1*α* were significantly higher in these tissues than those in para-tumour or normal tissues (Figure 1). Our results also demonstrated that K5 significantly inhibited the expression of HIF-1*α*, VEGF, and CD34 in xenograft gastric tumours in mice and in SGC-7901 tumour cells (Figure 2). Knockdown of HIF-1*α* led to the corresponding reduction of VEGF (Figure 3a). These data strongly supported the antiangiogenic effect of K5 in gastric tumours. This result is consistent with that of our previous studies, which showed that K5 suppressed the growth of hepatocellular carcinoma by targeting tumour angiogenesis.^14^

The hypoxic microenvironment contributes to tumour progression through the stabilization of the potent transcriptional factor HIF-1*α*. Under normoxia, HIF-1*α* is hydroxylated by the oxygen-sensitive PHDs, which leads to the binding of E3 ubiquitin ligase, VHL, to HIF-1*α* in a complex with elongin B, elongin C, and Cul2.^22, 23, 26^ When cells are exposed to a hypoxic microenvironment, this hydroxylation-mediated degradation pathway is blocked and results in HIF-1*α* translocation and accumulation in the nucleus, where it binds to the constitutively expressed HIF-1*β* to form a heterodimer and activates hypoxia-responsive genes, such as VEGF and Glu-1.^27, 28^ Consistent with previous results, HIF-1*α* accumulated under hypoxia (Figure 3d). As PHDs and VHL have an important role in the proteolytic degradation of HIF-1*α*, we next assessed which of these contributed to the K5-induced proteasomal degradation of HIF-1*α*. We found that K5 treatment under conditions of hypoxia upregulated VHL and downregulated HIF-1*α* (Figure 3d). The regulation of VHL by K5 in normoxia condition was measured; VHL was upregulated a little but much less than in hypoxia. The reason may come from the high constitutive expression of VHL under normoxia; the regulation under normoxia was not as obvious as under hypoxia. Our previous study showed similar results that K5 downregulated VEGF much significantly in hypoxia than in normoxia in the oxygen-induced retinopathy model.^29^ The common features of diabetic retinopathy and tumour microenvironment are hypoxia, therefore we speculate that the effects of K5 on VHL, HIF-1*α*, and VEGF are more significant and suitable for the treatment of the diseases with hypoxia microenvironment (Supplementary Figure S5). However, there was no change in the levels of PHD1 and PHD3. PHD2 demonstrated an increasing trend, but the increase was not statistically significant (Supplementary Figure S2), suggesting VHL, rather than PHDs, could be the target of K5. We further demonstrated that K5 facilitated the binding of HIF-1*α* to VHL and subsequent degradation of HIF-1*α* under hypoxia (Figure 3e). A recent study showed that SUMOylation of HIF-1*α*, which promotes its binding to VHL, leads to its ubiquitination and degradation via PHD-independent mechanism.^21^ Similarly, we observed that K5 treatment led to the decrease in HIF-1*α* by promoting HIF-1*α* SUMOylation and ubiquitination (Figure 3c). These results suggested that K5 stimulated the SUMOylation of HIF-1*α* and upregulation of VHL, which promoted HIF-1*α*/VHL binding and consequent ubiquitination and degradation of HIF-1*α*.

The receptor that mediates K5-induced VHL upregulation remains unknown. Several groups have independently demonstrated that VDAC serves as the receptor for K5 in human endothelial cells, 1-LN human prostate tumour cells, and retinal Müller cells.^18, 30^ Recently, our group showed that VDAC was upregulated by K5 in endothelial cells and served as the plasma membrane receptor to amplify the apoptotic effect of K5, acting as a ‘positive feed-back loop’.^17^ To investigate whether VDAC mediated the regulation of VHL by K5 in gastric cancer cells, we blocked VDAC expression by specific siRNA and the results showed that the effect of K5 on VHL was abolished (Figure 3g), which indicated that VDAC acts as the receptor in gastric cancer cells and mediates the antiangiogenesis effect of K5.

Antivascularization and antiangiogenesis therapies have been used to treat cancer. However, recent clinical observations indicated that antiangiogenesis agents such as Avastin and Sorafenib may have limited efficacy because of the induction of apoptosis resistance.^10, 25^ Interestingly, we found that K5 could induce apoptosis in SGC-7901 gastric cancer cells and gastric tumour xenografts in mice, in addition to exerting an antiangiogenesis effect (Figure 4). Downregulation of VEGF does not contribute to the pro-apoptotic effects of K5 because of the absence of VEGF receptors (Flt-1 and KDR) on SGC-7901 cells (Supplementary Figure S1). However, antiangiogenesis may lead to severe glucose and oxygen deprivation as well as GRP78 expression, which are HIF independent and closely related to apoptosis resistance.^10^ It is well known that GRP78 promotes tumour proliferation, survival, and metastasis.^11^ GRP78 overexpression confers resistance to a wide variety of chemotherapeutic agents in multiple tumour types, such as lung, bladder, stomach, breast, gastric, and epidermoid carcinoma. Knocking down GRP78 sensitizes the tumour cells to drug treatment.^12, 31, 32^ Our results showed that the level of GRP78 was elevated in all the 42 human gastric tumour specimens included in this study (Figure 1). Silencing GRP78 for 48 h resulted in extensive apoptosis of SGC-7901 cells and increased the levels of cleaved caspase-7 (Figures 5a and b), which indicates that GRP78 had a key role in the apoptotic process in gastric cancer. K5 could significantly downregulate GRP78 expression *in vivo* and *in vitro* (Figure 4), increasing the cleavage of caspase-7 (Figure 5c). These results are consistent with the recent reports that GRP78 protects cells from apoptosis during stress by binding to procaspase-7, blocking its activation, and decreasing stress-induced cellular apoptosis.^20, 30^

Zhang *et al.*^33^ and Song *et al.*^34^ indicated that activation of ERK by ER stress such as chronic hypoxia is necessary for the induction of GRP78, which protects gastric cancer cells against apoptosis.^33, 34^ As shown in Figures 6a and b, phosphorylation of ERK1/2 and expression of GRP78 were attenuated by K5 treatment and a specific inhibitor of ERK1/2. Neither JNK nor p38 was necessary for the K5-induced apoptosis (Supplementary Figure S4). Next we wanted to identify the receptor that mediated the downregulation of GRP78 by K5. As VDAC is a crucial regulator of apoptosis in many cancer types and mediates K5-induced apoptosis of endothelial cells,^17, 18, 35, 36^ VDAC was evaluated as the likely receptor of K5 in its regulation of GRP78. We used specific siRNAs to knockdown VDAC and found that the effects of K5 on GRP78 and p-ERK were abolished (Figures 6c and d), which highlighted the role of VDAC, as a critical receptor of K5, for both the antiangiogenesis and pro-apoptotic effects. GRP78 has also been identified as a receptor of K5,^19, 20^ and hence, whether GRP78 also acted as a receptor for K5 in our study needs to be investigated. Some studies also showed the co-localization of VDAC and GRP78 on the surface of 1-LN cells and endothelial cells.^30, 37^ It is possible that the downregulation of GRP78 by K5 results in the reduction of the cell surface GRP78, thus promoting the binding to VDAC, mediating the effects of antiangiogenesis, and pro-apoptosis.

**Figure 7:**
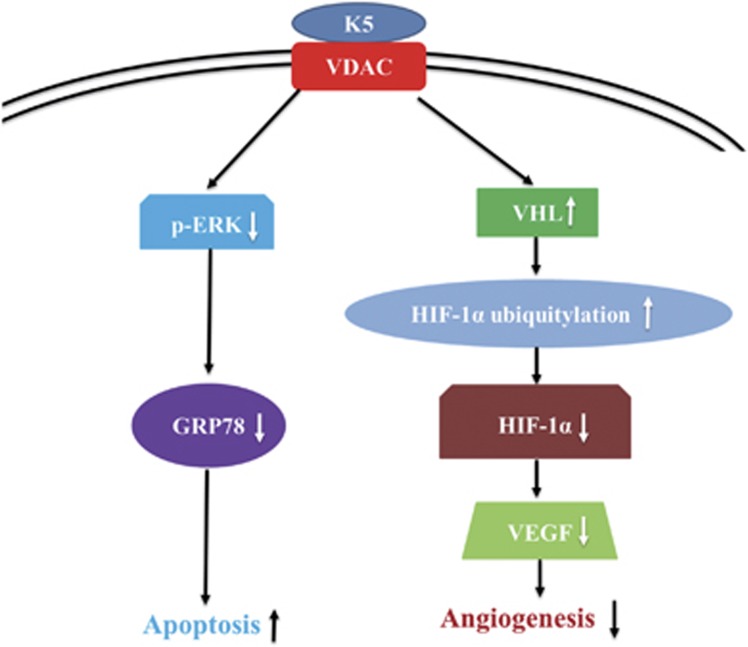
The schematic diagram of the signalling pathway of K5 in the regulation of tumour angiogenesis and tumour cell apoptosis

In summary, the present findings provide *in vivo* and *in vitro* evidence for the antiangiogenic and the pro-apoptotic effects of K5 in gastric cancer (Figure 7). The mechanism underlying these effects, downregulation of GRP78 and HIF-1*α* by K5, indicates that K5 may be a promising agent to counter the drug resistance of antiangiogenic therapies that result in severe glucose and oxygen deprivation.

## Materials and methods

### Chemicals

MG132 was obtained from Calbiochem (La Jolla, CA, USA), and U0126 was purchased from Cell Signalling Technology (Danvers, MA, USA).

### Cell culture

Malignant cell lines used in this study, human gastric carcinoma cells (SGC-7901 and BGC-823), were purchased from Sun Yat-sen University (Guangzhou, China) and maintained in Dulbecco’s modified Eagle medium (DMEM) supplemented with 10% foetal bovine serum (FBS, Gibco) at 37 °C in a humidified incubator in 5% CO_2_ atmosphere. To induce hypoxia, cells were placed in the Hypoxia Workstation containing 1% O_2_, 5% CO_2_, and 94% N_2_ (Binder, Bohemia, NY, USA).

### Human tissue samples

Tumour specimens from 42 patients who underwent surgical resection for gastric carcinoma of the stomach at the Third Affiliated Hospital of Sun Yat-sen University from 2001 to 2006 were used in this study. The tumour specimens were from 23 male patients and 19 female patients. The age distributions of these patients were: 11 patients ≤40 years, 14 patients 40–60 years, and 17 patients >60 years. Besides the 42 gastric carcinoma specimens, 17 para-tumour gastric specimens and 30 normal gastric tissue specimens were included in the study.

### Animal experiments

All the animal experiments in this study were conducted in strict accordance with the institutionally approved protocol according to the USPHS Guide for the care and use of laboratory animals, as well as the guidelines set forth in the Care and Use of Laboratory Animals by Sun Yat-sen University. Male 4-week-old athymic nude mice (BALB/c, nu/nu) were obtained from the Laboratory Animal Centre of Sun Yet-san University, Guangzhou, China. The animal license number is SCXK (YUE) 2007A015.

Subcutaneous implantation of SGC-7901 cells was performed by injecting 1 × 10^7^ cells into the lower right flank of athymic nude mice. When tumour volume reached about 50 mm^3^, the mice were randomized into two groups and received intraperitoneal injection of K5 or PBS. Human K5 was obtained as described previously.^14, 15^ The K5 group received K5 injection at the dose of 2.5 mg/kg per mouse, every 3 days, until the total dose reached 12.5 mg/kg. Control group was treated with the same volume of PBS. The tumours were measured on alternate days. Tumour volumes were calculated by the equation: volume=(length × width^2^) × 0.5. Tumours were collected 22 days after the injection of the cells and weighed.

### Immune staining assay

Immunocytochemistry and immunohistochemical analysis were performed as described earlier.^38^ Briefly, cells were seeded on gelatine-coated coverslips in six-well plates and treated with K5. The tissues were fixed in 4% paraformaldehyde solution and prepared as paraffin-embedded samples for immunohistochemical analysis. The samples were incubated with primary antibodies against CD34 from human epithelial cells (1 : 100, Dako, Copenhagen, Denmark), mouse CD34 (1 : 50, Abcam, Cambridge, UK), VEGF (1 : 100, Santa Cruz Biotechnology, Santa Cruz, CA, USA), HIF-1*α* (1 : 50, Santa Cruz), or GRP78 (1 : 100, Santa Cruz) at 4 °C overnight. Avidin–biotin–peroxidase was used for detecting immunolabelled cells and the sections were counterstained with haematoxylin. Primary antibodies were omitted in the staining of negative controls. Tumour MVD was quantified by the Weidner’s method.^39^ The images were captured by an Olympus microscope (Tokyo, Japan) at the magnification of × 400. ImagePro Plus 6.0 (Bethesda, MD, USA) was used to analyse the results.

### Quantitative RT-PCR

qRT-PCR was performed as described earlier.^40^ Briefly, total RNA was isolated using Trizol (Invitrogen, CA, USA) and reverse-transcribed (Takara, Shiga, Japan). GRP78 cDNA was amplified using primers 5′-GGAGAAGACTTTGACCAGCG-3′ and 5′-CTTTGGAATTCGAGTCGAGC-3′. Amplification reactions were performed with an initial denaturation step at 94 °C for 2 min, followed by 30 cycles consisting of a denaturation step at 94 °C for 5 min, an annealing step at 55 °C for 30 s, and an extension step at 72 °C for 1 min. To estimate the efficiency of cDNA synthesis, *β*-actin was used as a control. The PCR products were resolved on 1% agarose gels.

### Co-immunoprecipitation

For immunoprecipitations, 500 *μ*g SGC-7901 cell lysates were incubated with 4 *μ*g of anti-HIF-1*α* (Santa Cruz) over night at 4 °C, followed by the addition of 20 *μ*l Protein A or Sepharose G beads (Calbiochem) and incubated for 4 h at 4 °C. Immunoprecipitates were washed four times with lysis buffer, eluted with sample buffer, and immunoblotted.

### *In situ* detection of apoptosis by TUNEL staining

Frozen tumour sections (5 *μ*m thick) were analysed by TUNEL staining, using an *In Situ* Cell Death Detection Kit (Fluorescein; Roche Diagnostics, Indianapolis, IN, USA). Apoptotic cells appear as green-stained cells. The total number of cells, indicated by DAPI staining, was calculated by counting the cells in five arbitrarily selected fields at × 400 magnification. The apoptotic index was calculated using the equation, apoptotic cells × 200/total number of cells.

### Cell viability and apoptosis assays

SGC-7901 cells were seeded in 24-well plates at a density of 2 × 10^4^ cells per well and maintained in the growth medium until they reached 60% confluence. Cells were then treated with different concentrations (0, 80, 160, 320, 640, and 1280 nmol/l) of K5. Each treatment was set up in duplicate and incubated at 37 °C for 72 h under normoxia (21% O_2_) or hypoxia (1% O_2_). Cell viability was measured by MTT (Sigma, St. Louis, MO, USA) assay, according to the manufacturer’s protocol. Data represented absorbance and were expressed as the percentages of respective controls. For quantitative analysis of apoptosis, cells were treated as above for 48 h and then harvested for Annexin and propidium iodide staining, using the AnnexinV-FITC Apoptosis Detection Kit (Bender Med Systems, Vienna, Austria) according to the manufacturer’s instructions. Cells treated with 25 *μ*mol/l colchicine were used as positive control and those treated with PBS as negative control. The cells were subsequently counted using flow cytometry.

### siRNA transfection

Transfection was performed as described earlier.^41^ The sequences of shRNA targeting the 19-nucleotide segments were as follows: HIF-1*α* (sense, 5′-CCTATATCCCAATGGATGA-3′ antisense, 5′-CCTATATCCCAATGGATGA-3′, NM_001530); VHL (sense, 5′-CTGCCAGTGTATACTCTGA-3′ antisense, 5′-CTGCCAGTGTATACTCTGA-3′, NM_000551); GRP78 (sense, 5′–GGAGCGCATTGATACTAGA-3′ antisense, 5′-GGAGCGCATTGATACTAGA-3′, NM_005347); and VDAC (sense, 5′-AGATCAGCTTGCACGTGGACTGAAG-3′ antisense, 5′-TCGAAATCCATGTCGCAGCCCA-3′). The double-stranded oligonucleotides were cloned into an ApaI–EcoRI site in the pSilencer 1.0-U6 vector (Ambion, Austin, TX, USA). The plasmids were transfected into SGC-7901 cells using Lipofectamine 2000 (Invitrogen) for 6 h. After transfection, the cells were cultured for 18 h and then exposed to hypoxic atmosphere for the indicated times. The silencing efficiency was verified by western blotting analysis.

### Western blotting analysis

Western blotting analysis was performed as described earlier.^42^ Briefly, after separation on polyacrylamide gel, the proteins were transferred to PVDF membrane and subjected to western blotting analysis with antibodies against HIF-1*α* (1 : 1000, BD Biosciences; 1 : 500, Santa Cruz), VEGF (1 : 1000, Santa Cruz), VHL (1 : 1000, BD Biosciences, New York, USA), ubiquitin (1 : 1000, Santa Cruz), SUMO-1 (1 : 1000, Santa Cruz), GRP78 (1 : 2000, Santa Cruz), caspase-7 (1 : 500, Cell Signalling Technology), p-ERK (1 : 500, Cell Signalling Technology), ERK (1 : 1000, Cell Signalling Technology), and VDAC (1 : 1000, Cell Signalling Technology). Horseradish peroxidase-conjugated goat anti-mouse IgG or goat anti-rabbit IgG (1 : 3000 Sigma) were used as secondary antibodies. Protein bands were visualized using an enhanced Chemiluminescence Detection Kit (Pierce Biotechnology, Rockford, IL, USA) according to the manufacturer’s instructions. The same membrane was stripped and re-blotted with anti-*β*-actin antibody (1 : 5000, Sigma) for normalization.

### Statistical analysis

Data are presented as mean±S.E.M. from pooled data of 2–5 independent experiments in all figures and tables. Statistical analysis was performed by two-tailed independent Student’s *t*-test. *P*<0.05 was considered statistically significant.

## Publisher’s Note

Springer Nature remains neutral with regard to jurisdictional claims in published maps and institutional affiliations.

## Figures and Tables

**Table 1:** Expression of CD34, HIF-1*α*, VEGF and GRP78 in human gastric specimens

	**n**	**CD34**		**VEGF**			**Hif-1*****α***			**GRP78**		
		**Mean±S.E.M.**	**P**	**−**	**+**	**P**	**−**	**+**	**P**	**−**	**+**	**P**
Normal	30	18.18±4.344		25	5		29	1		22	8	
Para-tumour	17	23.35±9.861	<0.05^a^	4	13	0.73^a^	15	2	0.18^a^	10	7	0.31^a^
Tumour	42	34.94±11.64	<0.05^b^	10	32	<0.01^b^	5	37	<0.01^b^	11	31	<0.01^b^
			<0.05^c^,^b^			<0.0.092^c^			<0.01^c^			0.034^c^

‘−’, Negative; ‘+’, postive

a Para-tumour *versus* normal

b Tumour *versus* normal

c Para-tumour *versus* tumour
