# Supplementary material for: Plasminogen kringle 5 suppresses gastric cancer via regulating HIF-1α and GRP78
Source: Cell Death Dis. 2017 Oct 26;8(10):e3144–. doi: 10.1038/cddis.2017.528 (PMC5682690; doi:10.1038/cddis.2017.528)
Supplement: Supplementary Figure Legends [file cddis2017528x1.docx]

**Figure S1. No VEGF receptor exists on SGC-7901 cells**

Immunocytochemistry analysis of the expression of VEGF receptors (Flt-1 and KDR) in SGC-7901 cells under normoxia (1% O_2_) or hypoxic (21% O_2_) conditions for 24 hrs. N, normoxia; H, hypoxia.

**Figure S2. The influence of K5 on the expression of PHD1, PHD2 and PHD3 in SGC-7901 cells**

Western blot analysis of PHD1, PHD2 and PHD3 expressions in SGC-7901 treated with K5 for 24h under hypoxia. β-actin was used as a loading control.

**Figure S3. The effects of K5 on the proliferation and apoptosis of BGC-823 cells**

(A) BGC-823 cells were treated with K5 at concentrations indicated for 72 hrs under normoxia or hypoxia. The viable cells were quantified by MTT. ** P< 0.01.

(B) BGC-823 cells were treated with K5 (0, 160, 320, 640, 1280nM) for 48 hrs respectively, 25μM colchicine as positive control. Apoptotic cells were quantified by flow cytometry. Data are shown as mean ± SEM. * P< 0.05,** P< 0.01.

**Figure S4. The influence of K5 on P38 and JNK in SGC-7901 cells**

Western blot analysis of p-P38, P38, p-JNK and JNK expressions in SGC-7901 cells treated with K5 for 1h under hypoxia. β-actin was used as a loading control.

**Figure S5. The effect of K5 on VHL expression under normoxia in SGC-7901 cells**

Western blot analysis of HIF-1α, VEGF and VHL expressions in SGC-7901 cells treated with K5 for 8h (VHL) or 24h (HIF-1α, VEGF) under normoxia. β-actin was used as a loading control.
