# Supplementary figures and images for: Plasminogen kringle 5 suppresses gastric cancer via regulating HIF-1α and GRP78
Source: Cell Death Dis. 2017 Oct 26;8(10):e3144–. doi: 10.1038/cddis.2017.528 (PMC5682690; doi:10.1038/cddis.2017.528)

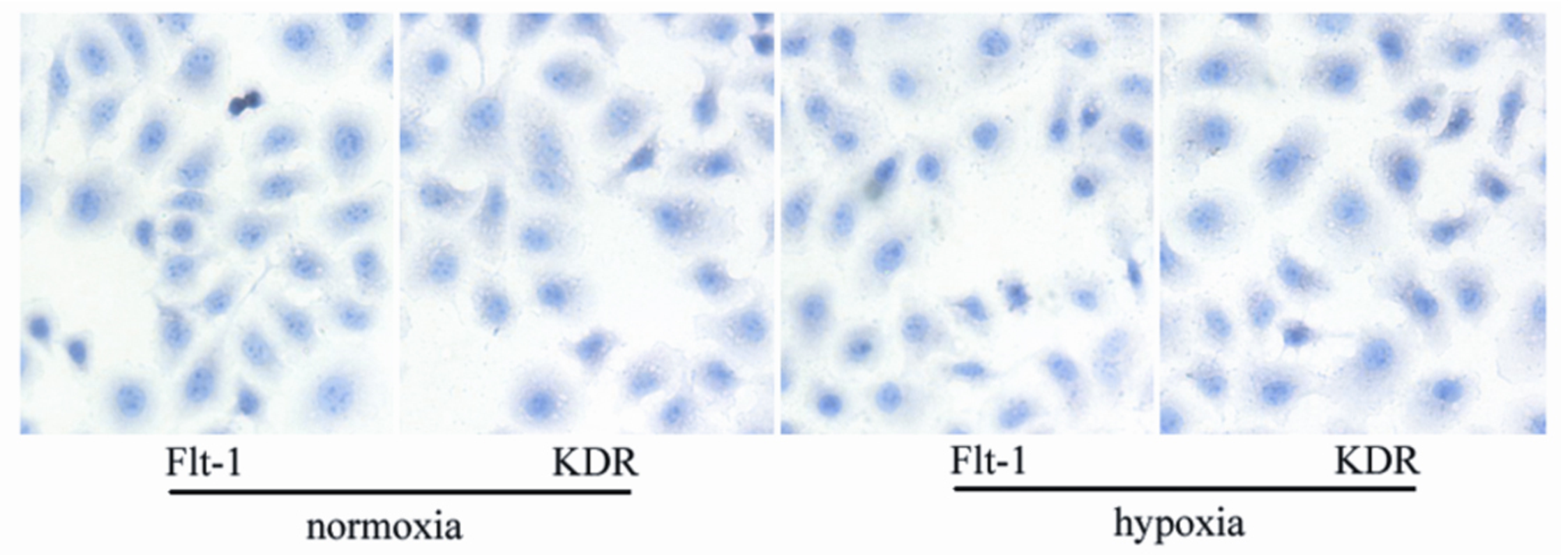

Supplement: Supplementary Figure S1 [file cddis2017528x2.tif]

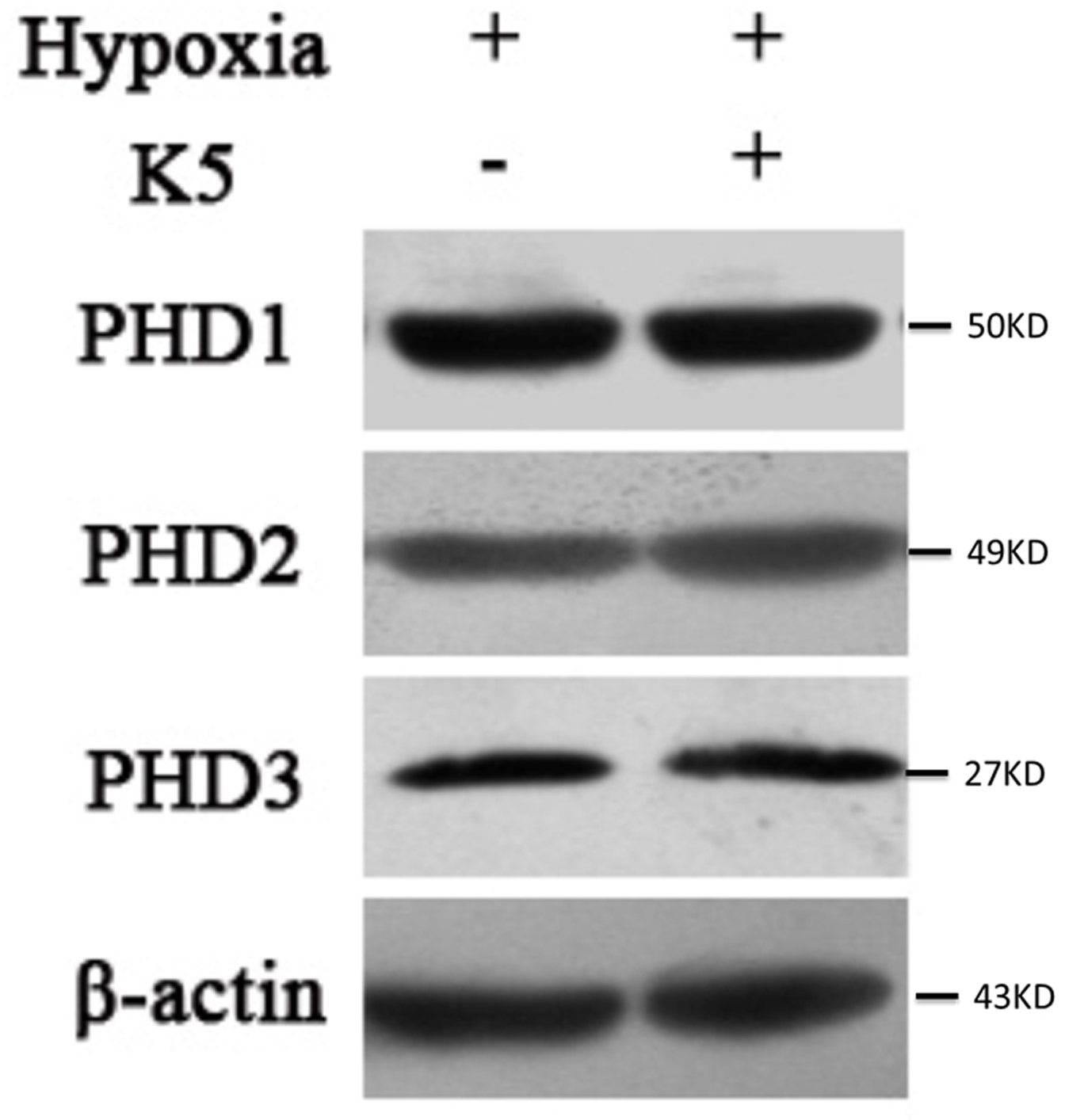

Supplement: Supplementary Figure S2 [file cddis2017528x3.tif]

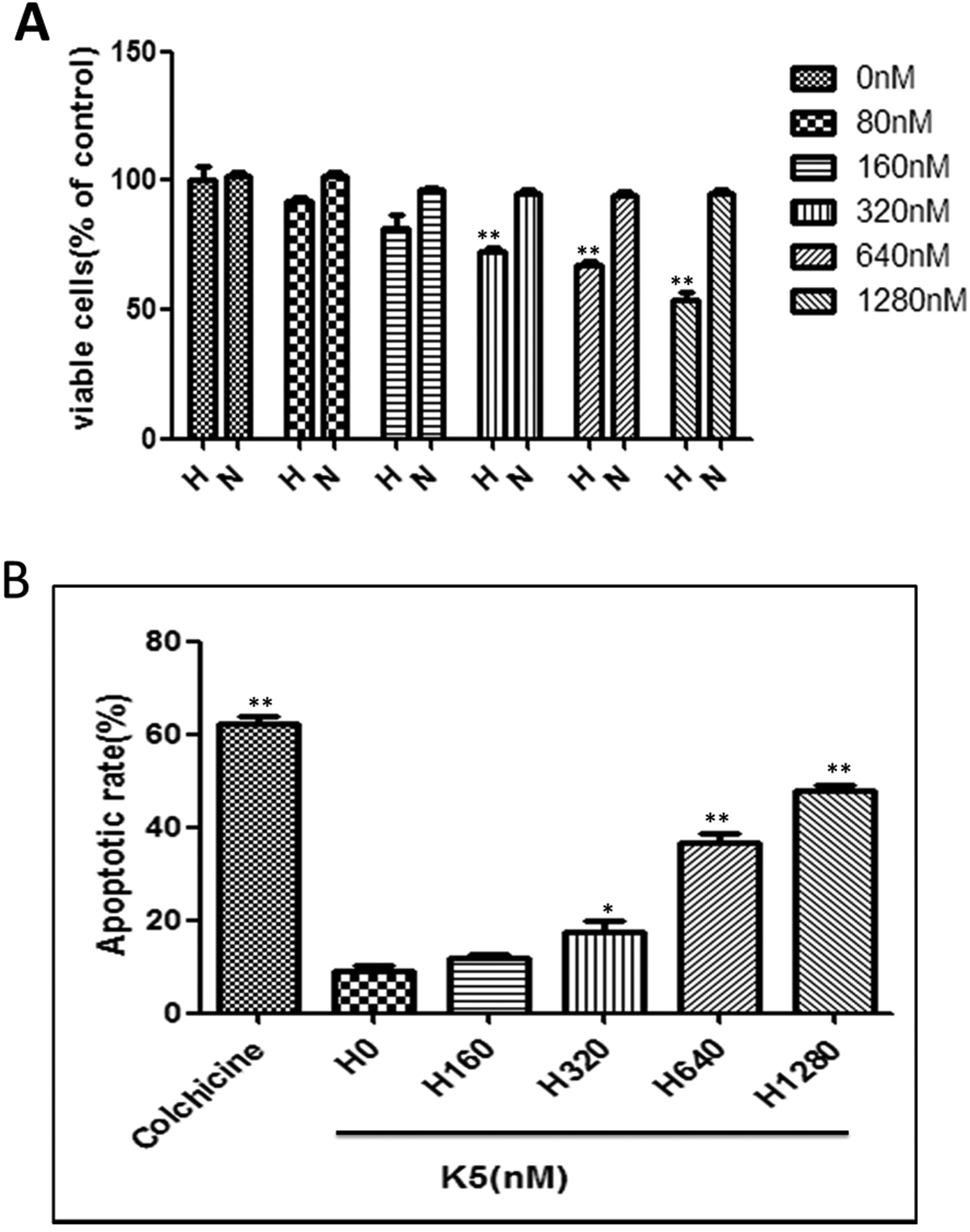

Supplement: Supplementary Figure S3 [file cddis2017528x4.tif]

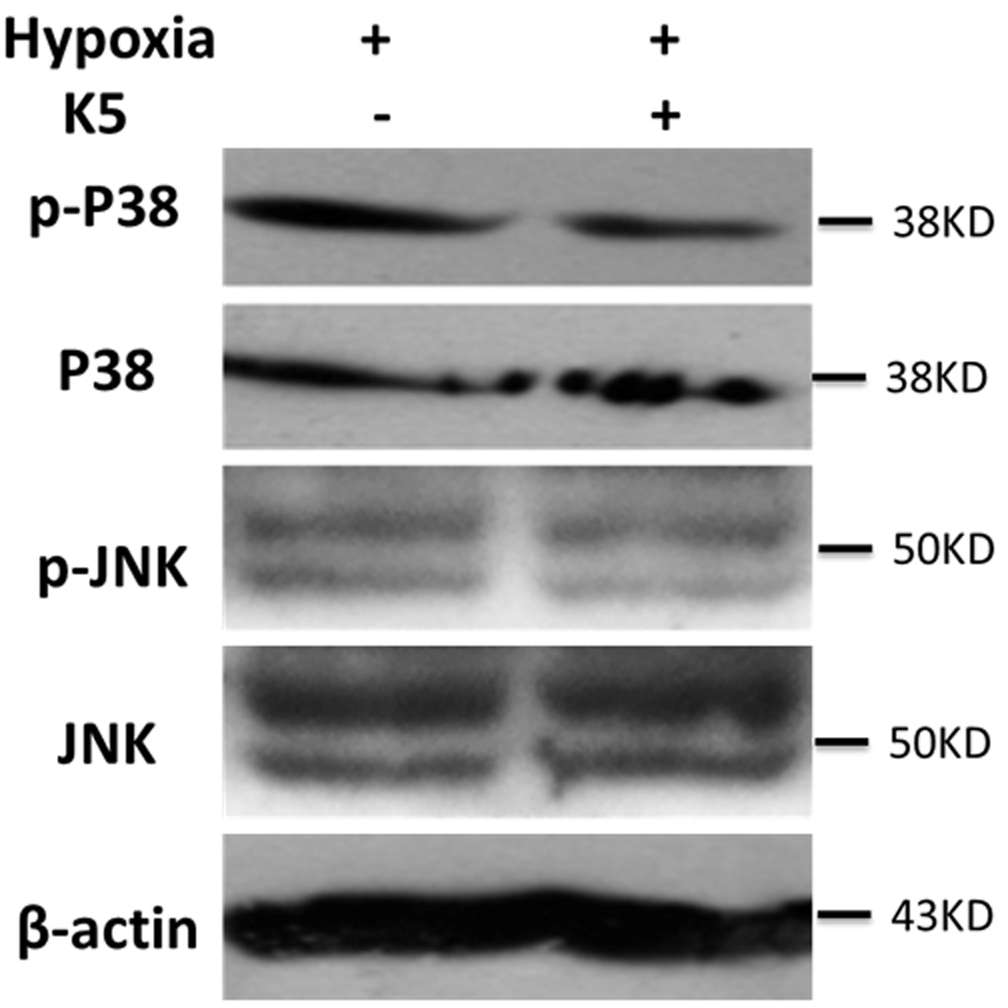

Supplement: Supplementary Figure S4 [file cddis2017528x5.tif]

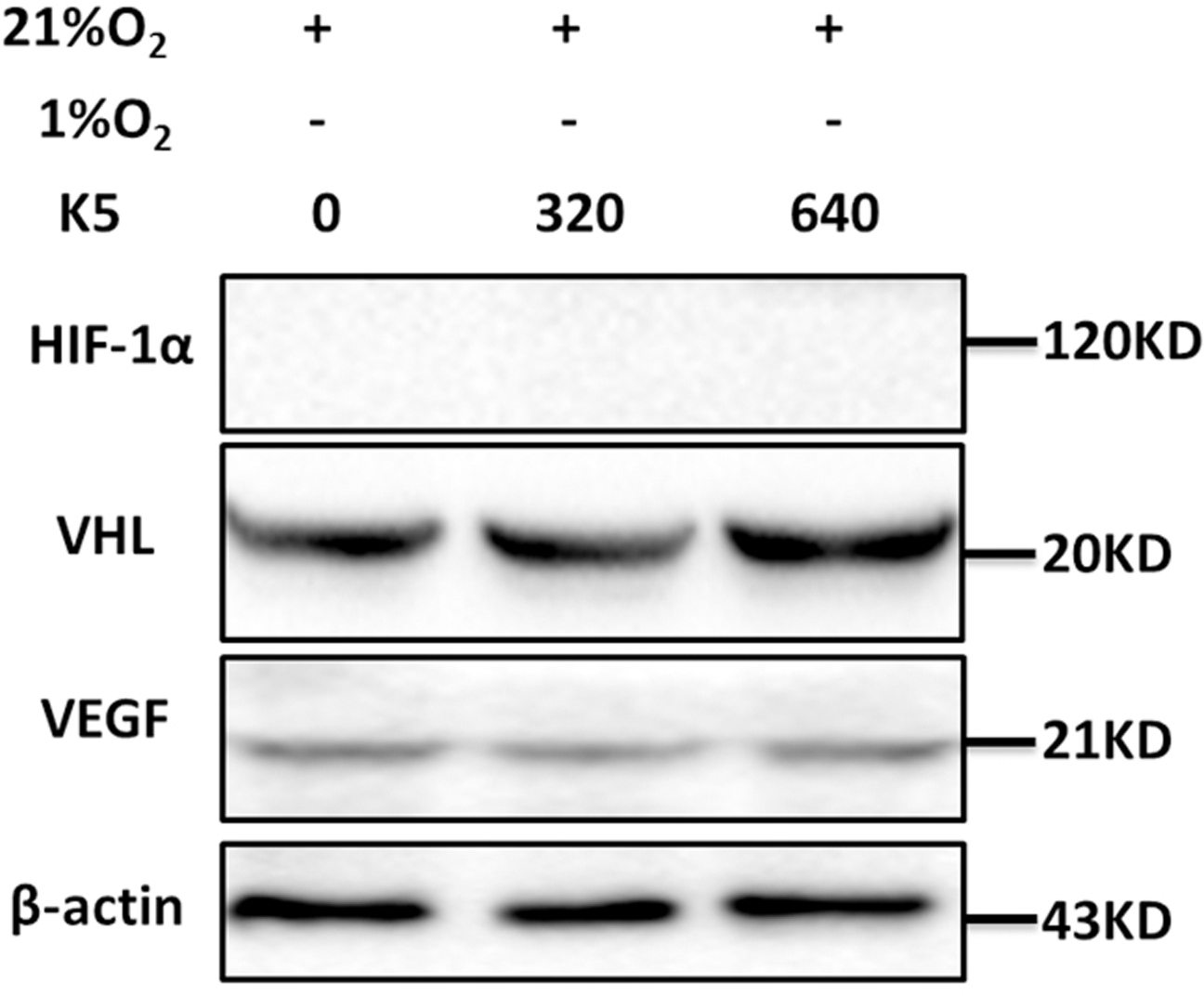

Supplement: Supplementary Figure S5 [file cddis2017528x6.tif]
